# Integrated Analysis of Expression Profile and Potential Pathogenic Mechanism of Temporal Lobe Epilepsy With Hippocampal Sclerosis

**DOI:** 10.3389/fnins.2022.892022

**Published:** 2022-06-16

**Authors:** Zhi-Bin Wang, Jian Qu, Zhuan-Yi Yang, Ding-Yang Liu, Shi-Long Jiang, Ying Zhang, Zhi-Quan Yang, Xiao-Yuan Mao, Zhao-Qian Liu

**Affiliations:** ^1^Department of Clinical Pharmacology, Hunan Key Laboratory of Pharmacogenetics, and National Clinical Research Center for Geriatric Disorders, Xiangya Hospital, Central South University, Changsha, China; ^2^Institute of Clinical Pharmacology, Engineering Research Center of Applied Technology of Pharmacogenomics, Ministry of Education, Central South University, Changsha, China; ^3^Department of Pharmacy, The Second Xiangya Hospital, Central South University, Changsha, China; ^4^Department of Neurosurgery, Xiangya Hospital, Central South University, Changsha, China; ^5^Department of Pharmacy, Xiangya Hospital, Central South University, Changsha, China

**Keywords:** hippocampal sclerosis, temporal lobe epilepsy, FGFR3, has-miR-486-5p, lnc-kCNH5-1

## Abstract

**Objective:**

To investigate the potential pathogenic mechanism of temporal lobe epilepsy with hippocampal sclerosis (TLE+HS) by analyzing the expression profiles of microRNA/ mRNA/ lncRNA/ DNA methylation in brain tissues.

**Methods:**

Brain tissues of six patients with TLE+HS and nine of normal temporal or parietal cortices (NTP) of patients undergoing internal decompression for traumatic brain injury (TBI) were collected. The total RNA was dephosphorylated, labeled, and hybridized to the Agilent Human miRNA Microarray, Release 19.0, 8 × 60K. The cDNA was labeled and hybridized to the Agilent LncRNA+mRNA Human Gene Expression Microarray V3.0,4 × 180K. For methylation detection, the DNA was labeled and hybridized to the Illumina 450K Infinium Methylation BeadChip. The raw data was extracted from hybridized images using Agilent Feature Extraction, and quantile normalization was performed using the Agilent GeneSpring. *P*-value < 0.05 and absolute fold change >2 were considered the threshold of differential expression data. Data analyses were performed using R and Bioconductor. BrainSpan database was used to screen for signatures that were not differentially expressed in normal human hippocampus and cortex (data from BrainSpan), but differentially expressed in TLE+HS’ hippocampus and NTP’ cortex (data from our cohort). The strategy “Guilt by association” was used to predict the prospective roles of each important hub mRNA, miRNA, or lncRNA.

**Results:**

A significantly negative correlation (*r* < −0.5) was found between 116 pairs of microRNA/mRNA, differentially expressed in six patients with TLE+HS and nine of NTP. We examined this regulation network’s intersection with target gene prediction results and built a lncRNA-microRNA-Gene regulatory network with structural, and functional significance. Meanwhile, we found that the disorder of FGFR3, hsa-miR-486-5p, and lnc-KCNH5-1 plays a key vital role in developing TLE+HS.

## Introduction

Epilepsy is a neurological disorder caused by abnormal discharge of the brain. It often manifests as a series of clinical symptoms such as transient or persistent limb twitches. There are about 50 million people with epilepsy worldwide, of which 80% are in developing countries. About 70% of epilepsy patients in the world receive effective treatment each year, but in countries with relatively backward development, more than 70% of epilepsy patients have not been effectively and adequately treated (Jessberger and Parent, 2021; Jobst and Cascino, 2015; Ben-Menachem, 2016; Keezer et al., 2016; Pitkanen et al., 2016; Elger and Hoppe, 2018; Spencer et al., 2018). Temporal lobe epilepsy with hippocampal sclerosis is a common type of temporal lobe epilepsy, which accounts for 70∼80% of temporal lobe epilepsy. It’s the most common type of intractable temporal lobe epilepsy in adults (Cendes et al., 2014; Gunn and Baram, 2017; Tai et al., 2018).

Epigenetic factors such as miRNA, lncRNA, and DNA methylation regulate gene expression and act as crucial regulators of neuron development, metabolism, and other processes (Alsharafi et al., 2015, 2016; Noebels, 2015; Henshall et al., 2016; Long et al., 2017; Xiao et al., 2017; Peng et al., 2018). Whole-genome miRNAs and lncRNAs have been analyzed to explore temporal lobe epilepsy with hippocampal sclerosis. Several miRNAs and lncRNAs have been identified with temporal lobe epilepsy with hippocampal sclerosis, including miRNA1234, miRNA-1281, miRNA-30c-1, miRNA-92b, miRNA-191, miRNA-1238. Long non-coding RNAs (lncRNAs) have been studied for a long time, and some new features have recently been discovered (Hormozdiari et al., 2015; Stone et al., 2018). The lncRNAs transcripts range in length from 200 nt to 100 kb. The expression profile of lncRNAs is tissue-specific and alters the biological function of lncRNAs in different cell types and diseases at various stages, which makes information on its location, binding site, and mode of action essential for studying the function of lncRNA. The function of lncRNA is closely related to the regulation of the network of mRNAs and miRNAs. However, almost no studies reported the association between lncRNAs and temporal lobe epilepsy with hippocampal sclerosis. Importantly, no one has linked miRNAs, lncRNAs, and mRNAs to study temporal lobe epilepsy with hippocampal sclerosis. In this consideration, we conducted a systematic study to understand the function of miRNAs, lncRNAs, and mRNAs. DNA methylation is an epigenetic alteration that involves the covalent attachment of a methyl group to the fifth carbon of cytosine residues. DNA methylation at the promoter region and throughout the genome can modify gene expression in a stable manner; this process has an impact on developmental and tissue-specific gene expression.

In this study, we will explore the expression profiles of microRNA/ mRNA/ lncRNA/ DNA methylation in brain tissues of temporal lobe epilepsy patients with hippocampal sclerosis and brain trauma and the potential pathogenic mechanism of temporal lobe epilepsy with hippocampal sclerosis.

## Materials and Methods

### Sample Collection and Clinical Characteristics

The Ethics Committee of Xiangya Hospital of Central South University approved the study (ethics approval number: CTXY-1300041-3). With informed consent, brain tissues were obtained retrospectively from Xiangya Hospital of Central South University (Changsha, Hunan, China). The subjects’ tissue was taken from the hippocampus of patients with temporal lobe epilepsy with hippocampal sclerosis (TLE+HS). Control tissue was taken from the normal temporal or parietal cortices (NTP) of patients undergoing internal decompression for traumatic brain injury (TBI). The study cohort consisted of 6 cases of TLE+HS and 9 cases of NTP. And we integrated the analysis based on the microarray results and subsequently collected the second batch of samples (15 cases of TLE+HS and 5 cases of NTP) for the validation of the microarray results. Tissues were collected and stored at –80°C until the microarray analysis.

### DNA and RNA Isolation and Expression Profiling

The subject sample was bisulfite converted using the Zymo EZ DNA Methylation Kit (Zymo Research) as directed. The Illumina 450k BeadChip was used to measure methylation. Total RNA was extracted using the TRIzol Reagent from Invitrogen, purified using NucleoSpin RNA clean-up, and quantified using a NanoDrop. The purity and integrity of the RNA were checked using gel electrophoresis. Total microRNA was purified using the MirVana miRNA Isolation Kit. To profile microRNA expression, the Human miRNA Microarray, Release 19.0, 8 × 60K (Agilent) was used, which contains 2,006 human miRNAs. To assess mRNA and lncRNA expression, the LncRNA+mRNA Human Gene Expression Microarray V3.0, 4 × 180K, which contains 28,844 human mRNAs and 37,581 human lncRNAs, was used. 200 ng of total RNA was labeled and hybridized using the miRNA Complete Labeling and Hyb Kit according to the manufacturer’s instructions (Agilent). The slides were scanned using an Agilent G2565CA Microarray Scanner, and the scanned images of the microarray were analyzed using Agilent Feature Extraction Software v10.7.

### Statistical Analysis

The degree of DNA methylation is represented by β value from 0 to 1. The original data was normalized and log 2-scale transformed by GeneSpring GX software. Probes on sex chromosomes and probes with a detection rate of less than 60% were filtered out.

The R package limma was used to perform comparisons between two groups (two-sided unpaired Student’s *t*-test). The Benjamini and Hochberg method was used to calculate the false discovery rate. The statistical significance cut-off values were adjusted *P*-value 0.05 and fold change 2. The pairwise combinations were filtered with a correlation coefficient less than −0.5, the correlation coefficient was adjusted with a false discovery rate.

### Correction of the Differential Expression Results Using the BrainSpan Database

The strategy of using normal temporal or parietal cortices from patients with TBI as a control group had some disadvantages. However, given ethical considerations (it is difficult to obtain normal hippocampal tissue as control), this was the last resort. Fortunately, the BrainSpan database provided a wealth of data on expression profiles of tissues in different parts of the human brain. Therefore, we tried to screen for signatures that were not differentially expressed in normal human hippocampus and cortex (data from BrainSpan) but differentially expressed in TLE+HS’ hippocampus and NTP’ cortex (data from our cohort). In this way, we tried to attenuate the interference of signatures expressed in different abundances in different brain regions in the results of this study. The paired samples from the BrainSpan database were compared using the paired samples *t*-test (difference normally distributed) or Wilcoxon test (difference non-normally distributed).

### Functional Analysis of Differentially Expressed mRNAs, miRNAs, and lncRNAs

To predict miRNA target mRNAs, the miRWalk database (a combination of TargetScan, miRDB, and miRTarBase) was used (Sticht et al., 2018). The ENCORI and LncBase v3 databases were also used to look for MiRNA target lncRNAs (Li et al., 2014; Karagkouni et al., 2020). A significantly negative Pearson’s correlation (*r* < −0.5) was used to establish correlated’ miRNA/mRNA or miRNA/lncRNA pairs.

Gene ontology (GO) analysis was performed on differentially expressed mRNAs (De_mRNAs) and the intersection of De_mRNAs and mRNA targets of differentially expressed miRNAs (De_miRNAs) to derive GO terms using the R package clusterProfiler (Wu et al., 2021). To identify potential TLE+HS pathogenic pathways, gene lists were analyzed using the Kyoto Encyclopedia of Genes and Genomes (KEGG) database.

The “Guilt by association” strategy was used to predict the prospective roles of each important hub mRNA, miRNA, or lncRNA (Ren et al., 2021; Urzua-Traslavina et al., 2021). The Pearson’s correlation coefficients between hub mRNA (or miRNA, lncRNA) and all mRNAs were used in Gene Set Enrichment Analysis (GSEA). The functional connections between hub mRNA (or miRNA, lncRNA) and KEGG terms were then calculated using GSEA.

## Results

### Clinical Characteristics of the Patients With Temporal Lobe Epilepsy With Hippocampal Sclerosis

We studied six patients with TLE+HS and nine with NTP. All patients were referred to the Xiangya hospital for their medical intractability. At the time of operation, the average age of patients with TLE+HS was 24 years old, ranging from 13 to 37. See Supplementary Table 1 for detailed clinical information on patients.

### Differential Expressed Signatures Between Patients With TLS+HS and Normal Temporal or Parietal Cortices

**FIGURE 1:**
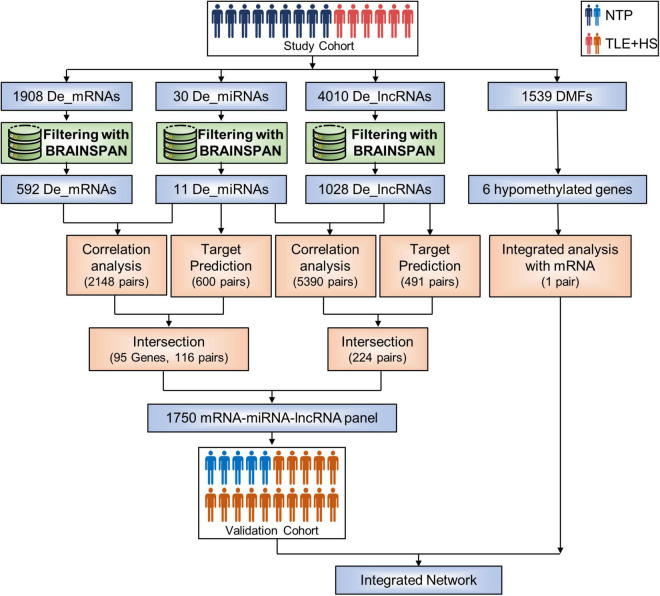
Sample groups and analysis process. Flowchart summarizing the study design and methods used. De_mRNAs, differential expressed mRNAs; De_miRNAs, differential expressed micro RNAs; De_lncRNAs, differential expressed long non-coding RNAs; DMFs, differentially methylated fragments; NTP, normal temporal or parietal cortices; TLE+HS, epilepsy with hippocampal sclerosis.

First, we identified 30 miRNAs, 1,908 genes, and 4,010 lncRNAs differentially expressed by the criteria of corrected *p*-value < 0.05 and fold change ≥ 2 in six patients with TLE+HS (Figure 1). The two-dimensional hierarchical clustering was performed using differentially expressed mRNAs, lncRNAs, and miRNAs. All heatmaps revealed clear separation of patients with TLE+HS and NTP tissues.

**FIGURE 2:**
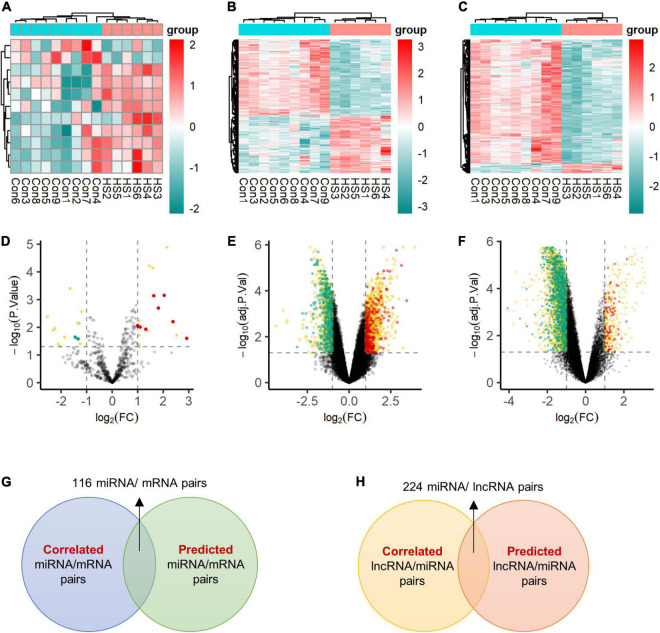
Expression profiles of differentially expressed transcripts. Two-dimensional hierarchical clustering of the significantly differential signatures of miRNA **(A)**, mRNA **(B)**, and lncRNA **(C)** in six patients with TLE+HS and nine of NTP (TLE+HS in blue, NTP in red). Signatures are in rows, samples are in columns. Volcano plots of differential expressed miRNA **(D)**, mRNA **(E)**, and lncRNA **(F)**. Red dots represent up-regulated signatures, green dots represent down-regulated signatures, yellow dots represent false positive differential expressed signatures filtered by BrainSpan database. **(G)** Intersect of correlated miRNA/mRNA pairs and predicted miRNA/mRNA pairs. **(H)** Intersect of correlated lncRNA/mRNA pairs and predicted lncRNA/mRNA pairs. Con, normal temporal or parietal cortices; HS, temporal lobe epilepsy with hippocampal sclerosis; FC, fold change.

The BrainSpan database was used to screen for signatures that were not differentially expressed in normal human hippocampus and cortex (data from BrainSpan), but differentially expressed in TLE+HS’ hippocampus and NTP’ cortex (data from our cohort). After filtering, a total of 11 miRNAs, 592 genes, and 1,028 lncRNAs were differentially expressed (Figures 2A–F).

We identified 2,148 pairs showing a strong inverse correlation between miRNA and gene expression by calculating the Pearson correlation coefficients (PCC) (PCC < −0.5). In addition, potential gene targets for each dysregulated miRNA were obtained by miRWalk^1^, which combined prediction results of different algorithms, including Targetscan, Mirdb, and Mirtarbase. At least one of these programs specifically identified the predicted gene targets. Finally, we identified 116 miRNA-gene co-expression pairs with PCC < −0.5 and *p*-value < 0.05 (Figure 2G).

In addition, for each dysregulated lncRNA, the PCC of its expression and that of each dysregulated miRNA were calculated. A 224 lncRNA-miRNA co-expression pairs were found by PCC < −0.5 or > 0.5 and *p*-value < 0.05 (Figure 2H).

### Gene Ontology and Pathway Analysis of Differential Expressed Signatures

**FIGURE 3:**
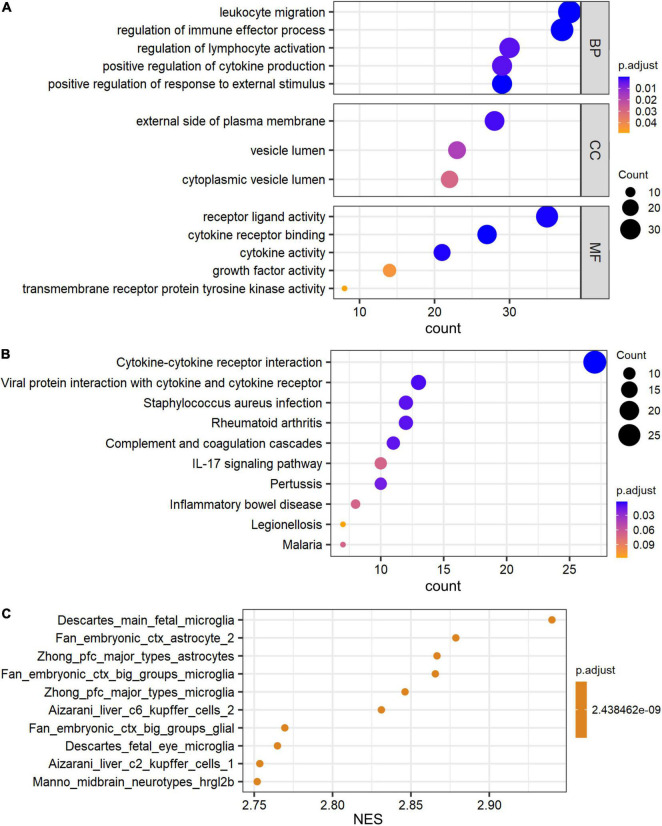
GO and pathway analysis of differential expressed signatures. Top 5 of GO_MF, GO_BP, GO_CC **(A)** and pathway **(B)** enriched in the differential expressed mRNAs between TLE+HS and NTP. **(C)** Gene set enrichment analysis (GSEA) terms for cell type signature gene sets were ordered according to their NES. GO, Gene ontology; KEGG, Kyoto Encyclopedia of Genes and Genomes; MF, molecular function; BP, biological process; CC, cellular component; TLE+HS, epilepsy with hippocampal sclerosis; NTP, normal temporal or parietal cortices.

The Gene Ontology (GO) and Kyoto Encyclopedia of Genes and Genomes (KEGG) pathway analyses were performed to determine the biological functions of these genes by R package ClusterProfiler. De_mRNAs were significantly enriched in GO terms, including leukocyte migration, external side of plasma membrane, and receptor ligand activity (Figure 3A). De_mRNAs were strongly enriched in KEGG pathways such as Cytokine-cytokine receptor interaction (Figure 3B). The differentially expressed signatures could, however, be due to differences in cell populations, as tissue with hippocampal sclerosis is expected to have fewer neurons and more astrocytes than the normal hippocampus. To answer this question, we performed a GSEA analysis of all mRNAs. The results are shown in Figure 3C and the top five significantly enriched cell types in order of normalized enrichment score (NES) are microglia or astrocyte. This suggests that the altered mRNA expression profile is likely due to astrocyte proliferation, which is one of the prominent pathological features of hippocampal sclerosis.

### Global DNA Methylation Analysis

**FIGURE 4:**
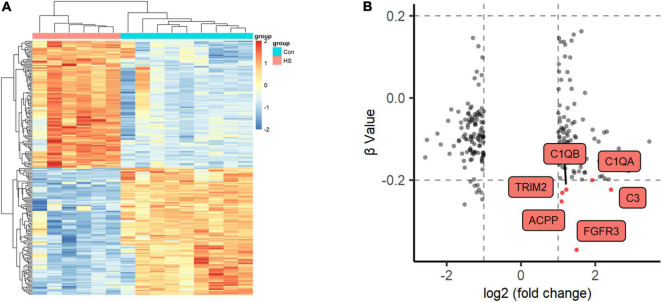
Methylation profiles of Global DNA. **(A)** Two-dimensional hierarchical clustering of the significantly differential methylation signatures between TLE+HS and NTP (TLE+HS in red, NTP in blue). **(B)** Nine quadrant diagram of the significantly differential methylation and mRNA signatures of Genes. TLE+HS, epilepsy with hippocampal sclerosis; NTP, normal temporal or parietal cortices.

**FIGURE 5:**
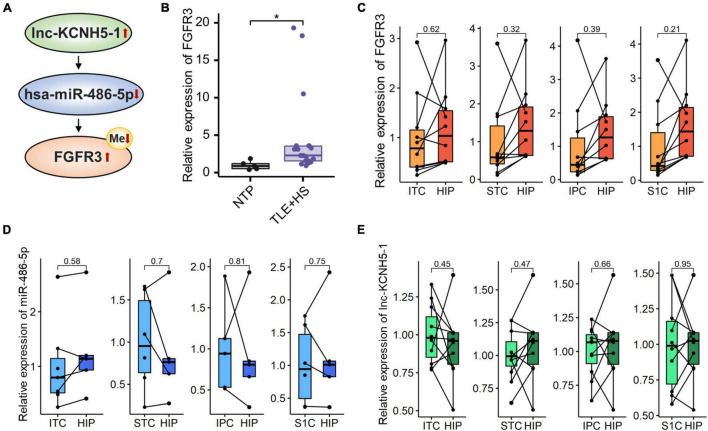
Networks of mRNA/miRNA/lncRNA related to TLE+HS. **(A)** Integrated regulation relationships of FGFR3, hsa-miR-486-5p, and lnc-KCNH5-1. **(B)** Validation of the FGFR3 mRNAs expression in another cohort (15 HS and 5 Con). Relative expression of **(C)** FGFR3, **(D)** hsa-miR-486-5P, and **(E)** lnc-KCNH5-1 in hippocampus tissues and different parts of temporal or parietal cortices in BrainSpan database (*n* = 10 pairs for FGFR3 and lnc-KCNH5-1, *n* = 3–5 pairs for hsa-miR-486-5p). NTP, normal temporal or parietal cortices; TLE+HS, temporal lobe epilepsy with hippocampal sclerosis; HIP, hippocampus tissue; ITC, inferolateral temporal cortex tissue; STC, posterior (caudal) superior temporal cortex tissue; IPC, posteroventral (inferior) parietal cortex tissue; S1C, primary somatosensory cortex (parietal cortex) tissue, *p < 0.05.

We also detected DNA methylation and found that six genes were hypomethylated (Figures 4A,B). At the same time, combined with the mRNA expression, we found that there was one gene FGFR3 with high mRNA expression that showed hypomethylation. FGFR3, with the most severe hypomethylation, was also highly expressed in TLE+HS. We speculate that abnormal methylation of FGFR3 may affect the gene’s function. Through integrated analysis of upstream lncRNA and miRNA of FGFR3, based on ceRNA theory, we constructed a gene interaction network to understand better their function and regulatory relationship (Figure 5A). Combined with the features of ceRNA theory, we hypothesized that the upregulation of lnc-KCNH5-1 reduces the expression of has-miR-486-5p by adsorbing it, which in turn inhibits the repressive effect of has-miR-486-5p on FGFR3, resulting in the upregulation of FGFR3 expression. Also, the reduction of FGFR3 methylation may contribute to the upregulation of FGFR3 expression.

### Validation of the Expression of the Critical mRNAs

We validated the expression of FGFR3 in the validation cohort (15 patients with TLE+HS and five with NTP). The results show that FGFR3 was highly expressed in TLE+HS in both our study and the validation cohort (Figure 5B).

To attenuate the interference of signatures expressed in different abundances in different brain regions in the results of this study, the expression profiles of tissues in different parts of the human brain from the BrainSpan database were used for filtering the results. Figures 5C–E showed the relative expression of FGFR3, has-miR-486-5p, and lnc-KCNH5-1 in the normal hippocampus and temporal or parietal cortices (*n* = 10 pairs for FGFR3 and lnc-KCNH5-1; *n* = 3–5 pairs for hsa-miR-486-5p). The results suggested that there was no difference in the expression of FGFR3, has-miR-486-5p, and lnc-KCNH5-1 in the normal hippocampus and temporal or parietal cortices. This indicates that the differential expression of the FGFR3, has-miR-486-5p, and lnc-KCNH5-1 between hippocampal tissue from TLE+HS patients and cortical tissue from NTP may not be due to tissue site differences.

### Function Prediction of FGFR3, Has-miR-486-5p, and lnc-KCNH5-1

**FIGURE 6:**
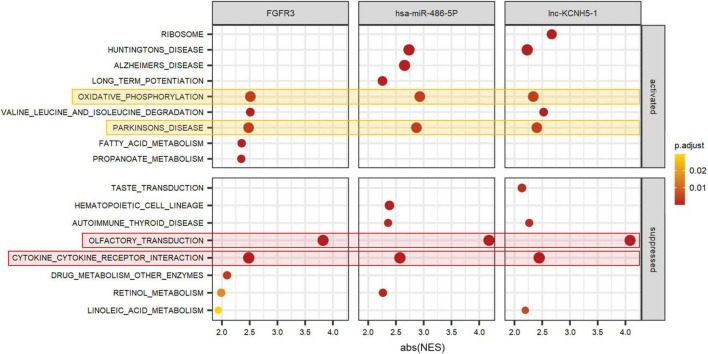
Function prediction of FGFR3, hsa-miR-486-5P, and lnc-KCNH5-1 by GSEA. GSEA terms were ordered according to their ranked abs (NES). GSEA, Gene set enrichment analysis; NES, normalized enrichment scores.

To better predict the gene function of FGFR3, has-miR-486-5p, and lnc-KCNH5-1, we calculated the correlation coefficients of the expression of FGFR3 (as well as has-miR-486-5p, and lnc-KCNH5-1) and all genes, respectively. GSEA analysis was performed using gene correlation coefficients. We found that the gene clusters that were significantly associated with the expression levels of these three signatures were focused on Olfactory transduction, Cytokine-cytokine receptor interaction, Oxidative phosphorylation, and Parkinson’s disease (Figure 6).

## Discussion

This study explored the expression profiles of microRNA/ mRNA/ lncRNA/ DNA methylation in brain tissues of temporal lobe epilepsy patients with hippocampal sclerosis and brain trauma and the potential pathogenic mechanism of temporal lobe epilepsy with hippocampal sclerosis. We found that the expressions of FGFR3, has-miR-486-5p, and lnc-KCNH5-1 were altered in TLE+HS patients.

Some other studies about the expression profile of temporal lobe epilepsy with hippocampal sclerosis, whereas most of them just analyzed only one type of expression profile (Guo et al., 2014; Kaalund et al., 2014; Alsharafi et al., 2016; Griffin et al., 2016). In a Czech study of 33 patients with TLE, 20 miRNAs differentially expressed in the hippocampus of epilepsy patients were identified (Bencurova et al., 2017). The control group used in this study was the hippocampus from postmortem. It is worth noting that miRNA-1260b and miRNA-339-5p were found both in the Czech research and ours. There are no reports on the association of these two miRNAs with epilepsy or hippocampal sclerosis disease. In Multiple System Atrophy, miRNA-339-5p was specifically downregulated (Su et al., 2022). MiR-339-5p has been linked to the NF-B and p53 signaling pathways, both of which are important for peripheral myelination (Wu, 2018).

FGFR3 is expressed during development in the brain and may play a function in nervous system development and hippocampus formation (Bernardo et al., 2021). Medial temporal lobe dysgenesis and seizures have been documented in Hypochondroplasia patients with an Asn540Lys mutation in the FGFR3 gene (Linnankivi et al., 2012; Okazaki et al., 2017).

GABRB3, one of the miR-486 target genes, encodes gamma-aminobutyric acid receptor subunit beta-3 (GABRB3). Gamma-aminobutyric acid is the nervous system’s primary inhibitory neurotransmitter. This gene’s missense mutation has been linked to a variety of neurological disorders, including epilepsy (Chen et al., 2020). This suggests that the function of miR-486 may be related to the transmission of neurotransmitters. Paschoal et al. pointed out that, miR-486–5p was associated with poor neurological admission status (Lopes et al., 2018).

No research on the function of lnc-KCNH5-1 has been reported. KCNH5 codes for a voltage-gated potassium channel. Members of this family regulate neurotransmitter and hormone release. The variants in KCNH5 were associated with epilepsy (Niday and Tzingounis, 2018). In epilepsy rats, upregulation of KCNH5 protein expression improved anxiety-like behavior and working memory (Liu et al., 2020).

Deficiencies in olfactory function may indicate the development or progression of neurological diseases. Structures involved in the mesial temporal lobe epilepsy were found in both the olfactory and limbic systems. Such structures establish a network that connects them, making them more vulnerable to excitatory harm from uncontrolled electrical activity (Aguilar Martínez et al., 2018). Patients with temporal lobe epilepsy due to hippocampal sclerosis had a basal olfactory dysfunction defined by impaired odor discrimination and recognition. Jovel et al. discovered a strong link between high seizure frequency and the examined olfactory tasks (Espinosa-Jovel et al., 2019). Neuroinflammation in the olfactory bulbs could be produced in a widespread manner throughout the brain, contributing to olfactory impairment in frontal lobe epilepsy patients (Mercado-Gómez et al., 2018).

Similar to our results, Wang et al. (2019) and Jiang et al. (2021) pointed out that there is a significant relationship between the Cytokine-cytokine receptor interaction pathway and the occurrence of epilepsy. Srivastava et al. (2017) findings implied that cytokine/chemokine regulatory networks in TLE+HS are altered differently. Reduced frequency of IFN-γ, TNF-α, IL-17, and IL-4 in CD8+ T lymphocytes of TLE+HS patients was observed (Rosa et al., 2016). Note that the occurrence of febrile seizures did not affect the expression of cytokines and chemokines in TLE+HS patients (Aulická et al., 2022).

There was a considerable enrichment for up-regulated genes involved in oxidative phosphorylation in TLE+HS in both our study and Griffin’s study (Griffin et al., 2016). Oxidative phosphorylation is a key player in energy conversion. Mitochondrial malfunction and oxidative stress have been linked to epilepsy and neurodegenerative disorders (Burtscher et al., 2015). Because mitochondrial oxidative phosphorylation is the primary source of ATP in neurons and mitochondria play a role in cellular Ca^2+^ homeostasis, they have the power to influence neuronal excitability and synaptic transmission (Kunz, 2002).

In our study, the gene clusters that were significantly associated with the expression levels of FGFR3, has-miR-486-5p, and lnc-KCNH5-1 also focused on Parkinson’s disease pathway. Parkinson’s disease is the second most prevalent neurodegenerative illness, featuring Lewy bodies and Lewy neurites as pathological hallmarks, as well as cell loss in the substantia nigra and other brain locations (Tolosa et al., 2021). Markopoulou et al. (2008) found substantial neuronal loss and gliosis in CA1 and subiculum with relative sparing of CA2 and the endplate in Parkinson’s disease patients, consistent with hippocampal sclerosis.

Taken together, we evaluated the microRNA/ mRNA/ lncRNA/ DNA methylation composition in hippocampal tissue samples from patients with TLE+HS (*n* = 6) and controls (*n* = 9). Additionally, *in silico* analyses of these signatures revealed that FGFR3, has-miR-486-5p, and lnc-KCNH5-1 and pathways associated with Cytokine-cytokine receptor interaction, Oxidative phosphorylation, and Parkinson’s disease may play a vital role in developing TLE+HS.

This study has some limitations. Although the high sensitivity and specificity of the microarray provide a high level of confidence in the current findings, it cannot rule out a false-negative rate due to the small sample size; therefore, these findings need to be experimentally validated in the future studies. Using the expression data of normal human hippocampus and cortex from the BrainSpan database to filter the results can only alleviate the possible interference due to the different tissue parts to a certain extent. At last, further investigation of differential expressed microRNA/ mRNA/ lncRNA/ DNA methylation, and their relevant targets might improve understanding and treatment of TLE+HS.

## Data Availability Statement

Publicly available datasets were analyzed in this study. This data can be found in the Gene Expression Omnibus (GEO) under accession GSE205661 (https://www.ncbi.nlm.nih.gov/geo/query/acc.cgi?acc=GSE205661).

## Ethics Statement

The studies involving human participants were reviewed and approved by the Ethics Committee of Xiangya Hospital of Central South University. Written informed consent to participate in this study was provided by the participants’ legal guardian/next of kin.

## Author Contributions

Z-BW conducted the experiments and wrote the manuscript. JQ, Z-YY, D-YL, and YZ approved the local ethical committees and sample collection. X-YM revised the manuscript. Z-QL and Z-QY approved the final manuscript. Z-QL, X-YM, and Z-QY contributed equally to this article. All authors contributed to the article and approved the submitted version.

## Conflict of Interest

The authors declare that the research was conducted in the absence of any commercial or financial relationships that could be construed as a potential conflict of interest.

## Publisher’s Note

All claims expressed in this article are solely those of the authors and do not necessarily represent those of their affiliated organizations, or those of the publisher, the editors and the reviewers. Any product that may be evaluated in this article, or claim that may be made by its manufacturer, is not guaranteed or endorsed by the publisher.

## Footnotes

1
http://mirwalk.umm.uni-heidelberg.de/
