# Supplementary material for: Integrated Analysis of Expression Profile and Potential Pathogenic Mechanism of Temporal Lobe Epilepsy With Hippocampal Sclerosis
Source: Front Neurosci. 2022 Jun 16;16:892022. doi: 10.3389/fnins.2022.892022 (PMC9243442; doi:10.3389/fnins.2022.892022)
Supplement: Supplementary file 1 [file Data_Sheet_1.PDF]

Table S1 | Clinical data of patients with or without TLE+HS.

Characteristic	Classes	TLE+HS	NTP
Gender	Male	3	7
	Female	3	2
Age	Age≤50	6	3
	Age＞50	0	6
Epilepsy	YES	6	0
	NO	0	9

[TLE+HS] Temporal lobe epilepsy with hippocampal sclerosis.

[NTP] Normal temporal or parietal cortices (NTP) of patients undergoing internal decompression for traumatic.
